# Phytochemical Characterization of *Mentha spicata* L. Under Differential Dried-Conditions and Associated Nephrotoxicity Screening of Main Compound With Organ-on-a-Chip

**DOI:** 10.3389/fphar.2018.01067

**Published:** 2018-09-28

**Authors:** Xian Li, Tian Tian

**Affiliations:** ^1^Chongqing Key Laboratory of Natural Product Synthesis and Drug Research, School of Pharmaceutical Sciences, Chongqing University, Chongqing, China

**Keywords:** spearmint, phytochemical profiles, nephrotoxicity, comparative metabolomics, organ-on-a-chip

## Abstract

Spearmint (*Mentha spicata* L.) is normally used as a vegetable flavoring herb. It also has several pharmacological activities against fever, cough, infection, and inflammation. The current study presents an untargeted comparative metabolomics approach utilizing HPLC-QTOF-MS high-throughput analytical technology to provide insights into the effect of the drying process on the examined spearmint species. To the best of our knowledge, this is the first report of compositional differences among fresh and dried spearmint leaves determined via a metabolomic approach to reveal that dried leaves are a better source of bioactive metabolites. The nephrotoxicity of kaempferol, a bioactive metabolite from spearmint, was further assessed with a kidney-on-a-chip. On the designed chip, a GelMA-based 3D culture platform mimics the microenvironment and basic functions of the kidney. In addition, the chip’s transparency allows for direct observation under an optical microscope. Treatment of human embryonic kidney cells with 30 μM of kaempferol for 12 h induced no obvious cell injury or apoptosis in the cells, on the basis of morphology, thus providing a proof-of-concept demonstration of kaempferol’s non-toxicity.

## Introduction

Spearmint (*Mentha spicata* L.) is an aromatic plant mainly found in Eurasia, Australia, South Africa, and China. Commonly, the fresh leaves are used as a raw vegetable or flavoring herb, whereas the dried leaves are traditionally used for herbal tea or medicine. Mint herbs have been reported to possess several biological effects, including antioxidant, anti-inflammatory, anticancer, and antimicrobial activities (Tang et al., 2016; Zaia et al., 2016; Wang et al., 2018). These biological activities of spearmint are significantly correlated with their total phenol flavonoid content (Manosroi et al., 2006; Santos et al., 2014).

In recent years, plant metabolomics has been successfully applied to metabolite profiling of different extract samples, thus bridging the knowledge gap between plant genotypes and phenotypes (De Vos et al., 2007; Creydt and Fischer, 2017). Progress in plant metabolomics techniques has made it possible to detect several hundred metabolites and to reliably compare differences and similarities between samples. Current metabolomics techniques utilize mass spectrometry (MS) coupled to gas or liquid chromatography, which achieves rapid metabolite analysis and has the advantages of allowing for high throughput and effective identification. Moreover, high-performance liquid chromatography quadrupole time-of-flight tandem mass spectrometry (HPLC-QTOF-MS) using electrospray ionization is a particularly well accepted platform for untargeted metabolite profiling in plant extracts (Su et al., 2018). In addition, to providing a comprehensive chemical profile of plants, chemometric methods are used for the classification and identification of metabolites and to define similarities and differences among varieties (Lubes and Goodarzi, 2017). There is, however, very little information reported on the metabolite differences between fresh and dried spearmint leaves. Besides, some scientific evidence indicates that several biochemical modifications and inter-conversions may occur during processing steps (Martins et al., 2016). Especially in traditional Chinese medicine, drying is considered to be the crucial step due to limiting enzymatic degradation and microbial growth while preserving the active principle content. Post-harvest fresh plant materials would exhibit a series of anti-dehydration mechanisms including production of related secondary metabolites at the early stage of dehydration (Li et al., 2012; Yuan et al., 2015). Given the beneficial health effects of spearmint, more detailed study of its phytochemical composition is worthwhile. The current study presents an untargeted comparative metabolomics approach utilizing HPLC-QTOF-MS high-throughput analytical technology together with principal component analysis (PCA) to provide insights into the effects of the drying process on the examined spearmint species.

Although a range of bioactive compounds in herbs and spices has been studied for antioxidant and anti-inflammatory properties *in vitro* and *in vivo* animal models, the present challenge lies in integrating this knowledge to ascertain whether these effects can be observed in humans. Since Huh et al. (2010) first described a lung-on-a-chip at the Wyss Institute, various organ-on-a-chip models have been developed to mimic the microenvironment and basic functions of tissues, and have become a novel drug screening platform. Those models yield reliable predictions and can be used to address problems that cannot ethically be investigated in humans, and for which available animal models have poor homology with humans. However, to date, there have been few reports on bioanalysis of active components from natural products through organ-on-a-chip systems. In this study, a kidney-on-a-chip was developed to investigate the nephrotoxicity of kaempferol, a bioactive metabolite from spearmint. Microfluidic techniques were introduced to mimic the continuous transport of nutrients and waste to and from the chip. To obtain a native extracellular matrix and functional tissue-tissue interfaces, GelMA, a product of gelatin methacrylate (Yue et al., 2015), was used for cell encapsulation, owing to its advantages of allowing for three-dimensional (3D) culturing and direct real-time visualization of cell conditions. The goals of this paper were (i) to investigate the phytochemical composition of both fresh and dried spearmint leaves and to understand the effect of drying treatment on metabolite profiles in spearmint by using HPLC-QTOF-MS, and (ii) to preliminarily develop a kidney-on-a-chip for nephrotoxicity testing of natural products.

## Materials and Methods

### Chemical and Reagent

Acetonitrile and methanol (HPLC grade) were purchased from Fisher Chemical. Methanol (HPLC grade) was purchased from Honeywell. DMEM, FBS, DPBS, penicillin–streptomycin, and trypsin-EDTA solution were purchased from Thermo Fisher Scientific.

### Leaf Extraction Procedure

The plant samples of *M. spicata* L. were purchased and collected from a well-known plant market in Shuyang, Jiangsu, China and identified by Xin Xu, in Heilongjiang University of Chinese Medicine. Fresh leaves were washed and ground with a pestle in a mortar under liquid nitrogen. The powder (250 mg) was homogenized with 2.5 ml 70% MeOH in an ultrasonic bath in ice water for 10 min (extracted for 1 min after paused for 1 min). Supernatant was obtained after centrifugation (10,000 rpm, 4°C) for 10 min three times. The supernatant was filtered through a 22-μm pore size filter, then mixed well. For dried leaf samples, fresh leaves were dried in an oven at 60°C for 20 h then ground with a pestle to powder and processed in the same manner as fresh samples. Six parallel experiments were performed for each group.

### High Resolution HPLC-MS Analysis

The extracts were diluted ten times before analysis. Chromatographic separation was performed on an XSELECT HPLC system (Waters) equipped with a CSH C18 column (100 × 2.1 mm, particle size, 3.5 μm). The analysis was carried out by applying the following binary gradient at a flow rate of 0.4 mL/min: 0–1 min, isocratic 90% A (water/formic acid, 99.9/0.1[v/v]), 10% B (acetonitrile/formic acid, 99.9/0.1 [v/v]); 1–24 min, linear from 10 to 40% B; 24–47 min, linear from 40 to 98% B. The injection volume was 10 μL (full loop injection). Eluted compounds were detected from m/z 100–710 in positive mode by using the following instrument settings: nebulizer and dry gas, nitrogen; gas temperature, 300°C; drying gas, 10 L/min; nebulizer, 40 psig; sheath gas temperature, 350°C; sheath gas flow, 12 L/min; Vcap, 4,000 V; nozzle voltage (Expt), 500 V. For analysis if biological variance, three biological replicates from each examined species were analyzed under the same conditions.

Raw data were processed in Agilent Mass Hunter Qualitative Analysis software. The total 36 HPLC-QTOF-MS data files (36 samples × 37 peak area variables) were collected and submitted to Progenesis QI (Waters) for PCA. A ChemSpider^1^ library search was also used to check the mass fragmentation behavior of all compounds.

### 3D Cell Culturing

Gelatin was dissolved in DPBS solution, and the beaker walls were covered with aluminum foil to provide darkness. The mixture was stirred until completely dissolved in a 60°C water bath. Methacrylic anhydride was then added dropwise into the beaker protected from light. The reacted solution was diluted with DPBS in a water bath at 50°C. The diluted homogenous solution was transferred to a dialysis membrane (8000–14,000/44 mm) and placed in sterile distilled water for 1 week of dialysis, with the water changed periodically. The dialyzed jelly was dispensed into a centrifuge tube and, after ultraviolet sterilization, was cryopreserved to a solid at -80°C. It was freeze-dried to a spongy consistency.

The photoinitiator (PI) was dissolved in DPBS solution in a beaker covered with aluminum foil, then heated in an oven until it completely dissolved. GelMA was mixed with PI/PBS solution and heated in an oven until it completely dissolved. Human embryonic kidney cells (293T cells) were counted after trypsinization. The supernatant was aspirated after centrifugation, and DPBS solution was added to adjust the cell suspension concentration to 10^7^ cells/mL. Human embryonic kidney cells were encapsulated in 5 wt% gelatin methacryloyl (GelMA), loaded into the bottom chamber of a microbioreactor, and subsequently photo-crosslinked against the mask to generate a 3D human primary kidney organoid. The cured hydrogels were washed with 5, 3, and 1 mL DPBS. Then the appropriate amount of culture medium was added, and the samples were returned to a 37°C CO_2_ incubator.

The kidney-on-a-chip system was filled with DMEM with a microinjection pump (Harvard Apparatus, PHD/Ultra) with a flow rate of 0.1 mL/min. After the bubbles were drained, the flow rate was adjusted to 8 μL/min, and the system was placed in an incubator for 1 h, to achieve continuous and stable chemical exchange through the microfluidic system. The 293T cells encapsulated in GelMA were cultured separately for 24, 48, 72, and 96 h, then stained with Trypan Blue and compared with cells cultured in dishes. Kaempferol powder was dissolved in DMEM at a concentration of 30 μmol/L and transferred with a flow rate of 8 μL/min.

## Results

### Total Compound Content of Spearmint (*Mentha spicata* L.)

**FIGURE 1:**
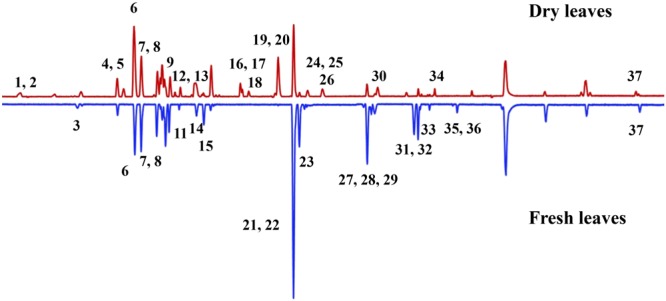
Base peak chromatogram data of both dried and fresh leaves. The main spearmint metabolites identified in the extract and peak numbers are the same as in **Table 1**.

**FIGURE 2:**
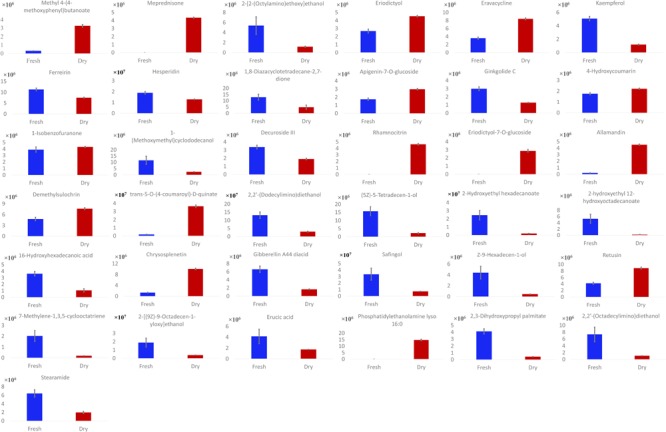
Quantification of compounds in dried and fresh spearmint.

Phytoconstituents of *M. spicata* L. were analyzed via reversed-phase HPLC-QTOF-MS, using a gradient mobile phase consisting of acetonitrile and water. Complete elution of metabolites was achieved within approximately 45 min. A total of 37 metabolites were detected, as listed in **Table 1**. Metabolite assignment was performed by comparing retention times (Rt), MS data (accurate mass, isotopic distribution, and fragmentation pattern) based on ChemSpider library search and previously reported findings of the compounds detected (Cao et al., 2011; Cirlini et al., 2016) (**Figure 1**). As summarized in **Figure 2**, there were six metabolites found in only dried leaves, including meprednisone (peak 2), rhamnocitrin (peak 16), eriodictyol-7-*O*-glucoside (peak 17), allamandin (peak 18), *trans*-5-*O*-(4-coumaroyl)-D-quinate (peak 20), and phosphatidylethanolamine lyso 16:0 (peak 34). In contrast, there were only two metabolites, 2-hydroxyethyl hexadecanoate (peak 23) and 2-hydroxyethyl 12-hydroxyoctadecanoate (peak 24), found only in fresh leaves. Notably, the spearmint metabolites were extracted with menthol aqueous solution, owing to its high permeability to cells and favorable compound solubility. Because a series of tests indicated that the metabolites eluted mainly under aqueous acetonitrile solution between 20 and 50%, the initial ratio of acetonitrile was set as 10% with a lower flow rate and linear slope (**Supplementary Figures S2, S3**).

**Table 1:** List of identified metabolites of spearmint (*Mentha spicata* L.).

No.	m/z	Rt(min)	Compound	MS^2^	Adducts	Formula	Source
1	209.1164	5.44	Methyl 4-(4-methoxyphenyl)butanoate	/	M+H	C_12_H_16_O_3_	D, F
2	411.1592	5.44	Meprednisone	291.1014, 250.1055, 151.9926	M+K	C_22_H_28_O_5_	D
3	218.2110	6.84	2-[2-(Octylamino)ethoxy]ethanol	/	M+H	C_12_H_27_NO_2_	D, F
4	289.0697	10.16	Eriodictyol	153.0180	M+H	C_15_H_12_O_6_	D, F
5	597.1786	10.16	Eravacycline	289.0692, 153.0180, 85.0285, 71.0491	M+K	C_27_H_31_FN_4_O_8_	D, F
6	287.0542	10.89	Kaempferol	153.0175	M+H	C_15_H_10_O_6_	D, F
7	303.0852	12.94	Ferreirin	177.0543, 153.0181	M+H	C_16_H_14_O_6_	D, F
8	611.1934	12.94	Hesperidin	153.0132, 85.0284, 71.0492	M+H	C_28_H_34_O_15_	D, F
9	453.3422	13.25	1,8-Diazacyclotetradecane-2,7-dione	114.0916	2M+H	C_12_H_22_N_2_O_2_	D, F
10	433.1108	13.34	Apigenin-7-O-glucoside	271.0585	M+H	C_21_H_20_O_10_	D, F
11	463.1212	13.76	Ginkgolide C	301.0691	M+Na	C_20_H_24_O_11_	D, F
12	163.0390	15.13	4-Hydroxycoumarin	/	M+H	C_9_H_6_O_3_	D, F
13	135.0441	15.13	1-Isobenzofuranone	117.0336, 89.0385, 63.0233	M+H	C_8_H_6_O_2_	D, F
14	246.2424	15.62	1-(Methoxymethyl)cyclododecanol	106.0862, 88.0757, 70.0650	M+NH_4_	C_14_H_28_O_2_	D, F
15	593.1837	16.47	Decuroside III	447.1262, 285.0746	M+Na	C_26_H_34_O_14_	D, F
16	323.0534	18.13	Rhamnocitrin	295.0589, 163.0386, 133.0282	M+Na	C_16_H_12_O_6_	D
17	489.0786	18.13	Eriodictyol-7-O-glucoside	311.0531, 283.0587	M+K	C_21_H_22_O_11_	D
18	331.0796	18.72	Allamandin	316.0562, 288.0612	M+Na	C_15_H_16_O_7_	D
19	341.0644	20.78	Demethylsulochrin	295.0582, 221.0588, 165.0694, 133.0284	M+Na	C_16_H_14_O_7_	D, F
20	361.0903	20.81	*Trans*-5-O-(4-coumaroyl)-D-quinate	331.0434, 182.9920	M+Na	C_16_H_18_O_8_	D
21	274.2734	21.76	2,2’-(Dodecylimino)diethanol	106.0864, 88.0758, 70.0652, 57.0697	M+H	C_16_H_35_NO_2_	D, F
22	230.2475	21.87	(5Z)-5-Tetradecen-1-ol	230.2469, 57.0696	M+NH_4_	C_14_H_28_O	D, F
23	318.2996	22.17	2-Hydroxyethyl hexadecanoate	256.2628, 88.0758, 70.0651	M+NH_4_	C_18_H_36_O_3_	F
24	362.3257	22.5	2-Hydroxyethyl 12-hydroxyoctadecanoate	332.0462, 300.2881	M+NH_4_	C_20_H_40_O_4_	F
25	290.2682	22.68	16-Hydroxyhexadecanoic acid	242.2468, 88.0758	M+NH_4_	C_16_H_32_O_3_	D, F
26	375.1060	23.79	Chrysosplenetin	342.0716	M+H	C_19_H_18_O_8_	D, F
27	387.1788	26.59	Gibberellin A44 diacid	105.0701	M+Na	C_20_H_28_O_6_	D, F
28	302.3048	26.79	Safingol	106.0865, 88.0757, 70.0622, 57.0696	M+H	C_18_H_39_NO_2_	D, F
29	258.2787	26.96	Z-9-hexadecen-1-ol	/	M+NH_4_	C_16_H_32_O	D, F
30	359.1114	27.54	Retusin	343.0794, 298.0822, 162.0675	M+H	C_19_H_18_O_7_	D, F
31	119.0859	30.01	7-Methylene-1,3,5-cyclooctatriene	/	M+H	C_9_H_10_	D, F
32	330.3359	30.27	2-[(9Z)-9-octadecen-1-yloxy]ethanol	88.0758	M+NH_4_	C_20_H_40_O_2_	D, F
33	356.3511	31.02	Erucic acid	131.0014, 88.0755, 55.0540	M+NH_4_	C_22_H_42_O_2_	D, F
34	476.2743	31.27	Phosphatidylethanolamine lyso 16:0	335.2564, 62.0603	M+Na	C_21_H_44_NO_7_P	D
35	353.2676	32.63	2,3-Dihydroxypropyl palmitate	261.2202, 81.0702	M+Na	C_19_H_38_O_4_	D, F
36	358.3668	32.92	2,2’-(Octadecylimino)diethanol	106.0861, 88.0754	M+H	C_22_H_47_NO_2_	D, F
37	284.2941	45.41	Stearamide	102.0915	M+H	C_18_H_37_NO	D, F

*D and F represent the dried and fresh leaves.*

### Identification of Flavonoids

**FIGURE 3:**
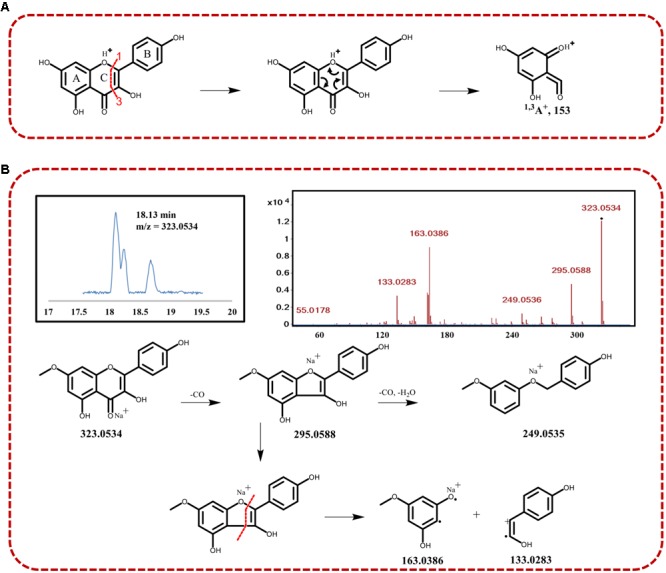
**(A)** Schematic diagram of the retro Diels–Alder (RDA) fragmentation at the 1–3 bonds of the C-ring of kaempferol under collision-induced dissociation. **(B)** Identification of compound 16 with TIC, MS2 assays, and the fragmentation process.

On the basis of total compound content, spearmint has a high content of kaempferol and quercetin derivatives, thus providing nutritional value and antioxidant ability. The presence of the skeleton of kaempferol yielded an RDA fragmentation pattern under positive collision-induced dissociation. The characteristic fragment was ^1.3^A^+^ (m/z approximately 153), through cleavage at the bonds between positions 1 and 3 in the C-ring, as shown in **Figure 3A**. Using QTOF-MS, we observed RDA fragmentation from compounds 4, 6, 7, and 8 (m/z 153.0180, 153.0175, 153.0181, and 153.0132, respectively). These compounds appeared to have the same skeleton of as kaempferol, and we were able to discriminate among them on the basis of the other different fragments. In addition, in the presence of methylation of C_7_-OH, the RDA fragments of ^1.3^A^+^ (expected m/z 167) were significantly inhibited, and collision-induced dissociation (CID) fragmentation was more obvious. The signal peak of m/z 167 allowed for identification of methylation of C_7_-OH, differentiating RDA and CID fragmentation. We were also able to distinguish different flavonoids, because CID fragmentation was often accompanied by a decreased intensity of RDA fragments. For example, in the identification of rhamnocitrin (compound 16), with the C_7_-OH of rhamnocitrin methylated, we observed the m/z 163.0386 fragment from CID fragmentation, rather than ^1.3^A^+^ (m/z 167) from the RDA. In general, some fragments are derived from neutral/radical loss during CID fragmentation, such as carbonyl and water. Thus, the other ion signals, such as m/z 295.0588 ([M+Na-CO]^+^) and m/z 249.0535 ([M+Na-2CO-H_2_O]^+^) were found as well. On the basis of chemical compound reference data, chemical structure was identified, and the fragmentation process is illustrated in **Figure 3B**.

### Multivariate Data Analysis of Fresh or Dried Spearmint Leaves Analyzed via HPLC-ESI-QTOF

**FIGURE 4:**
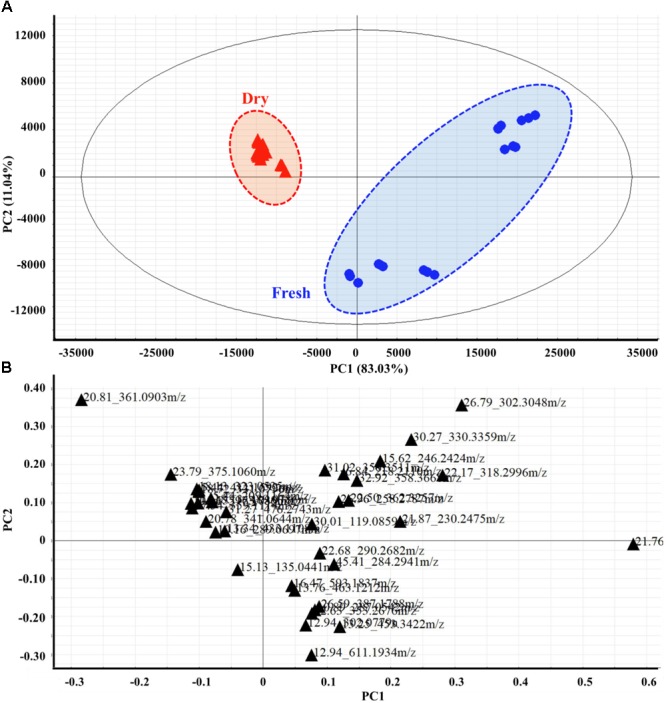
Two-dimensional PCA score plots **(A)** and loading plots **(B)** derived from HPLC-MS spectra data sets of fresh and dried leaves, showing that the metabolomes were clearly different between the two groups. Score plots are described by two vectors of principal component 1 PC1 = 83.03% and PC2 = 11.04%. Each metabolite is denoted by its Rt (min) – Mass pair in loading plots.

An HPLC-ESI-QTOF chromatographic matrix of phenolic compounds (36 samples × 37 peak variables) was subjected to PCA to compare the discrimination of the spearmint sample metabolites with the results obtained in the previous sections. As demonstrated on the PC1 versus PC2 plot (**Figure 4A**), the spearmint samples were well-separated according to fresh or dried condition. The score plot indicated that the samples clustered in two separate groups. The dried samples formed a tight group in the PC1–PC2 plane, and both PCs clearly contributed to the discrimination. For the leaves of all samples, the resulting PC1 (83.03% variance explained) versus PC2 (11.04% variance explained) plot showed that the first two PCs accounted for more than 90% of the total variance. Discrimination of the fresh from the dried samples was possible along PC1. The objects from fresh samples overlapped mostly with positive scores on PC1, whereas the objects from dried samples were tightly concentrated with negative scores on this PC. When the scores on these two PCs were observed in the PC1–PC2 plane, the two groups were reasonably separated.

The metabolite loading plot (**Figure 4B**) indicated the most important components with respect to scattering behavior. The plot demonstrated that PC1 was strongly influenced by the 2,2′-(dodecylimino) diethanol (peak 21, Rt_m/z: 21.76_274.2734) loading, with positive values, appearing most distant, and *trans*-5-*O*-(4-coumaroyl)-D-quinate (peak 20, Rt_m/z: 20.81_361.0903) loading showing negative values. PC2 was strongly influenced by the positive loading of safingol (peak 28, Rt_m/z: 26.79_302.3048) and 2-[(9Z)-9-octadecen-1-yloxy] ethanol (peak 32, Rt_m/z: 30.27_330.3359), but hesperidin (peak 8, Rt_m/z: 12.94_611.1934) loading correlated with negative values.

### Kidney-on-a-Chip for Nephrotoxicity Test

**FIGURE 5:**
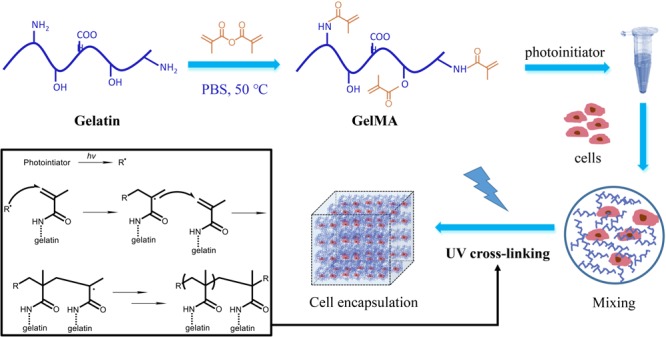
The scheme of GelMA preparation and cell encapsulation. Gelation involved methacrylation to form GelMA, and cross-linking under PI catalysis and exposure of UV light. Cells are encapsulated in the hydrogel and cultured in 3D.

GelMA is prepared from gelation and comprises normal collagens derived from animal skin, modified with methacrylate groups (**Figure 5**). Our results indicated that the presence of PI in GelMA hydrogel along with the photolithography process had minimal effects on endothelial cell survival, and the cells grew normally in hydrogels after 4 days (**Supplementary Figure S4**).

**FIGURE 6:**
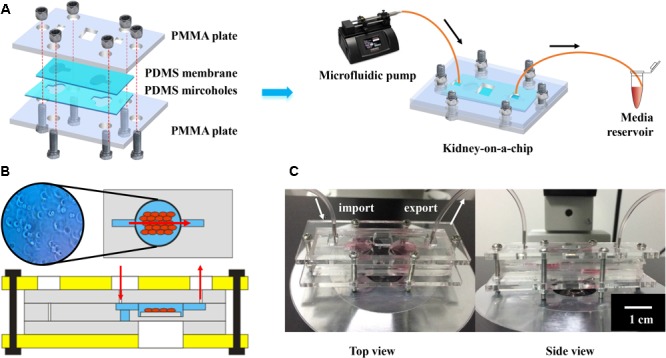
Characterization of kidney-on-a-chip for nephrotoxicity testing: **(A)** The schematic illustration of the chip, with PDMS membranes for scaffolding, PMMA plates for fixing and continuous medium flow from the microfluidic pump for nutriment exchange. **(B)** The internal structure and cell conditions of the kidney-on-chip and **(C)** photographs showing top view and side view of the chip.

As illustrated in **Figure 6A**, the bioreactor was designed to possess a chamber embedded in a pair of polydimethylsiloxane (PDMS) pieces, which were sandwiched between two rigid supports made of polymethyl methacrylate (PMMA). These assembly units constituted a sandwich arrangement completely sealed with a set of screws and bolts. PMMA is the typical material used for covering, owing to its firmness and rigidity. PDMS was used as the scaffold material in this chip, because of its advantages of transparency, high flexibility and sealing. The pair of micro-featured PDMS layers together formed the bioreactor chamber at a thickness of approximately 2 mm when closed, which were connected with inlet and outlet ports on the two sides. The main chamber was square (9 × 9 mm^2^) (**Figure 6B**). Finally, the bioreactor featured a circular opening in the center of the PMMA support at the bottom to enable direct microscopic monitoring of the morphology of the GelMA-based microtissue without a need for disassembling the bioreactor (**Figure 6C**). To evaluate the direct pharmacological damage to the engineered micro-kidney, we continuously perfused the scaffold with 30 μM of kaempferol added into the medium for a pharmacokinetic cycle of 12 h. After staining with Trypan Blue solution, the living cells were with light blue, and the dead cells were dark blue, as compared with the background. Observed under a light microscope, the cells were irregularly round and distributed individually without any black debris. The cytoplasm was translucent, and the nucleus was darker, results comparable to normally growing cells (**Figure 6B**).

## Discussion

This study provides detailed metabolite profiling of spearmint and an exact comparison of metabolite composition between dried and the fresh *M. spicata* L. samples, via HPLC-ESI-QTOF. According to detected phytoconstituents of spearmint, the metabolites belonged to various classes, including 17 aliphatic compounds and their derivatives (e.g., 16-hydroxyhexadecanoic acid and erucic acid), nine flavonoids and their glycosides (e.g., kaempferol and hesperidin), three phenolic acids and their derivatives (e.g., *trans*-5-*O*-(4-coumaroyl)-D-quinate), one polyketone, three terpenes, two coumarins and their glycosides, one arene derivative and one steroid (**Supplementary Figure S1**). These compounds were mostly of higher polarity. It is generally assumed that the drying process removes the water from leaves and increases the concentrations of metabolites. However, our research showed that some compounds (compounds 6, 11, and 16) had comparable abundance in fresh and dried samples. Furthermore, compounds 23 and 24 were much more abundant in fresh leaves than dried leaves, thus indicating that some biochemical modifications and interconversions actually occur during the drying processing steps. In fact, the diversity in chemical composition between two groups was derived from not only the abundance but also the variety of the ingredients. From the metabolite profiling, dried leaves were found to contain more flavones and other non-volatile secondary metabolites regarded as the bioactive compounds, whereas fresh leaves contained more primary metabolites.

Given the complexity of spectral data, the multivariate data analyses used for extracting useful information and various methods of data processing in chemometrics are increasingly being used in herbal drug analysis (Wang et al., 2017; Montuschi et al., 2018). In the present study, PCA was applied to better visualize the subtle similarities and differences between the fresh and dried spearmint samples. These chemometric analysis suggested that the composition of the non-volatile compounds in the spearmint samples from dried and fresh leaves differed substantially. The details of distinction have been shown in the results section. It should be noted that as fresh samples are processed by liquid nitrogen, the powder cannot act as dried samples and would undergo wetting more readily, thus resulting fresh samples clustering less tightly on the plot, whereas the dried samples overlapped in a tight cluster around the origin. The similarities and differences in the metabolite groups between dried and fresh products are concordant with dried products being used more often as raw materials in actual applications.

Spearmint has a high content of polyphenols, such as flavonoids, thus providing anti-inflammatory and anti-oxidant ability. Flavonoids in spearmint generally occur as sugar conjugates, principally as *O*-glycosides, and are the most abundant metabolite class. Full scan MS analysis identified nine flavonoid peaks (4, 6, 7, 8, 10, 16, 17, 26, and 30), mostly as flavonol glycosides, on the basis of their mass data, and kaempferol and quercetin derivatives were abundant. Because the fragmentation of the aglycone moiety often follows certain rules, such as retro Diels–Alder (RDA) fission, the flavonoids have other characteristic fragments resulting from the different substituents on the skeleton, and the structures could consequently be identified. MS/MS was performed to aid in structural elucidation, and the sugar type in *O*-glycosides was determined from the respective loss of 162, 146, and 132 amu corresponding to glucose, deoxyhexose, and pentose, respectively (de Villiers et al., 2016). In addition, the disaccharide and oligosaccharide were deduced from monosaccharide loss. In this work, MS spectral interpretation led to the identification of eriodictyol (m/z 289.0697, [M+H]^+^, C_15_H_12_O_6_, peak 4) and its conjugate, eriodictyol-7-O-glucoside (m/z 489.0786, [M+K]^+^, C_21_H_22_O_11_, peak 17). Whereas the adduct of compound 17 changed from K^+^ to H^+^, the m/z was approximately 451 amu. The loss of 162 amu corresponded to glucose, and the exact type was easily identified from the MS/MS pattern. Similarly, the difference between apigenin-7-*O*-glucoside (m/z 433.1108, [M+H]^+^, C_21_H_20_O_10_, peak 10) and its fragment of m/z 271.0585 resulted from the loss of a glucose moiety (-162 amu). In MS/MS analysis, the nature of sugars was revealed from elimination of the sugar residue, that is, 162 amu (glucose). After the full complement of bioactive compounds in *M. spicata* L. has been elucidated, studies to better explain their medicinal use or food properties will be necessary.

Flavonoids were recognized as the main bioactive metabolites among the non-volatile compounds in spearmint. Notably, six of them were present in both fresh and dried leaves, such as kaempferol, a flavonoid widely present in fruits and vegetables. Previous studies have shown that adequate dietary kaempferol is beneficial for human health (Mecocci et al., 2014). However, although kaempferol has strong effects in modulating apoptosis (Dang et al., 2015), whether it might cause damage to normal human cells must be evaluated. In this study, a kidney-on-a-chip was developed to assess the nephrotoxicity of kaempferol. This GelMA-based 3D culture platforms can provide a tissue-like environment because of their potential to mimic the natural extracellular matrix of kidney tissues. We developed an innovative resealable microbioreactor for perfusion culture to support the long-term viability of human embryonic kidney cells encapsulated in GelMA and *in situ* observation of the 3D cultured micro-kidney constructs, by modifying recently published protocols (Zhang et al., 2017).

As a biocompatible hydrogel, GelMA not only non-cytotoxic and biodegradable to cells but also consists of intrinsic arginine, glycine, and aspartate sequences. Thus, it is a superior material for mimicking extracellular matrix interactions *in vitro* (Madl and Heilshorn, 2018; Yue et al., 2018). To perform cell encapsulation in 3D human embryonic kidney cell culture, we introduced micropatterning by exposing the proper mixture of GelMA and PI solution to UV light, to form covalent cross-links and polymerization. This cell-encapsulated mircropatterned network recapitulated the structural and functional complexity of organs. The micropatterned constructs were developed with 5% GelMA with high methacrylation (70% wt), owing to its mechanical robustness and suitability for micropatterning (Nichol et al., 2010).

Thus, this chip provided hydraulic tightness and easy access to the hydrogel scaffold-containing bioreactor and also made analysis rapid and straightforward. The circulating medium pumped with a syringe pump can transport nutrients or waste as well as various metabolites generated by human embryonic kidney cells to the reservoir via integrated outlet ports, thereby simulating the material exchange process of cells in human bodies. In addition, owing to the chip’s transparency, it is possible to observe the morphology of human embryonic kidney cells directly under an optical microscope. On the basis of morphology, treatment with 30 μM of kaempferol did not induce cell injury or apoptosis in the human embryonic kidney cells, thus providing a proof-of-concept demonstration of kaempferol non-toxicity via this kidney-on-a-chip platform. The results were in agreement with published studies on animal and other *in vitro* models (Ahlenstiel et al., 2003; Park et al., 2009).

## Conclusion

In this work, we performed non-volatile metabolite profiling of both fresh and dried spearmint leaves by using HPLC-ESI-QTOF for untargeted large-scale metabolite analysis. A total 37 metabolites were identified, and the main differences were in metabolite abundance and the variety. The results indicated that the basic metabolite profiles can be altered by drying process to obtain more secondary metabolites, such as flavones, which possessed potential bioactivities. The PCA revealed the details of compositional differences and similarities in the spearmint samples. The major differentially present metabolites between fresh and dried leaves included rhamnocitrin, eriodictyol-7-*O*-glucoside, 2-hydroxyethyl hexadecanoate, and 2-hydroxyethyl 12-hydroxyoctadecanoate, thus illuminating why dried leaves are more useful and effective than fresh leaves. In addition, GelMA was prepared and applied to cell encapsulation for 3D culture, and kidney-on-a-chip was used in the first demonstration of a kaempferol nephrotoxicity test. This study extended the exploration of dehydration mechanisms of spearmint and introduced a new system based on organ-on-a-chip for bioanalysis of active principle content in traditional Chinese medicine.

## Author Contributions

TT conceived and designed research. XL and TT conducted experiments and analyzed data. TT and XL wrote the manuscript. All authors read and approved the manuscript.

## Conflict of Interest Statement

The authors declare that the research was conducted in the absence of any commercial or financial relationships that could be construed as a potential conflict of interest.

## Footnotes

1 http://www.chemspider.com
