# Supplementary material for: Phytochemical Characterization of Mentha spicata L. Under Differential Dried-Conditions and Associated Nephrotoxicity Screening of Main Compound With Organ-on-a-Chip
Source: Front Pharmacol. 2018 Sep 28;9:1067. doi: 10.3389/fphar.2018.01067 (PMC6190883; doi:10.3389/fphar.2018.01067)
Supplement: Supplementary file 1 [file Data_Sheet_1.pdf]

Phytochemical Profiles and Nephrotoxicity Screening of *Mentha spicata* L. Subjected to Different Drying conditions: Comparative Metabolomics and Organ-on-a-chip

Xian Li, Tian Tian*

Chongqing Key Laboratory of Natural Product Synthesis and Drug Research, School of Pharmaceutical Sciences, Chongqing University, Chongqing 401331, China

Corresponding Author.

Tian Tian., PhD., Assistant Professor, School of Pharmaceutical Sciences, Chongqing University, Chongqing 401331, China

Email.: tiantian@cqu.edu.cn

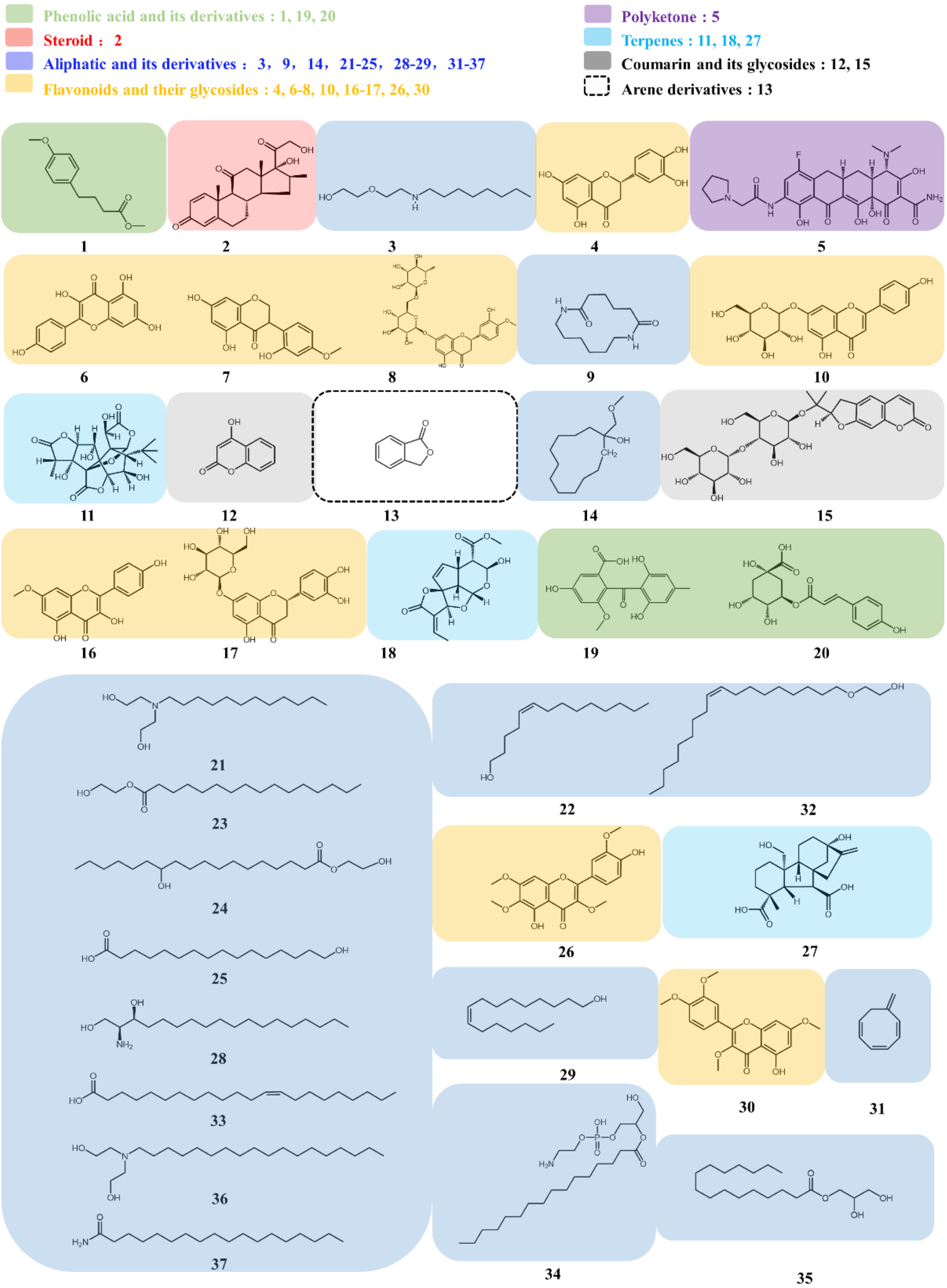

Fig. S1 The structures of identified metabolites, including 3 phenolic acid and its derivatives, 1 steroid, 17 aliphatic and its derivatives, 9 flavonoids and their glycosides, 1 polyketone, 3 terpenes, 2 coumarin and its glycosides and 1 arene derivatives.

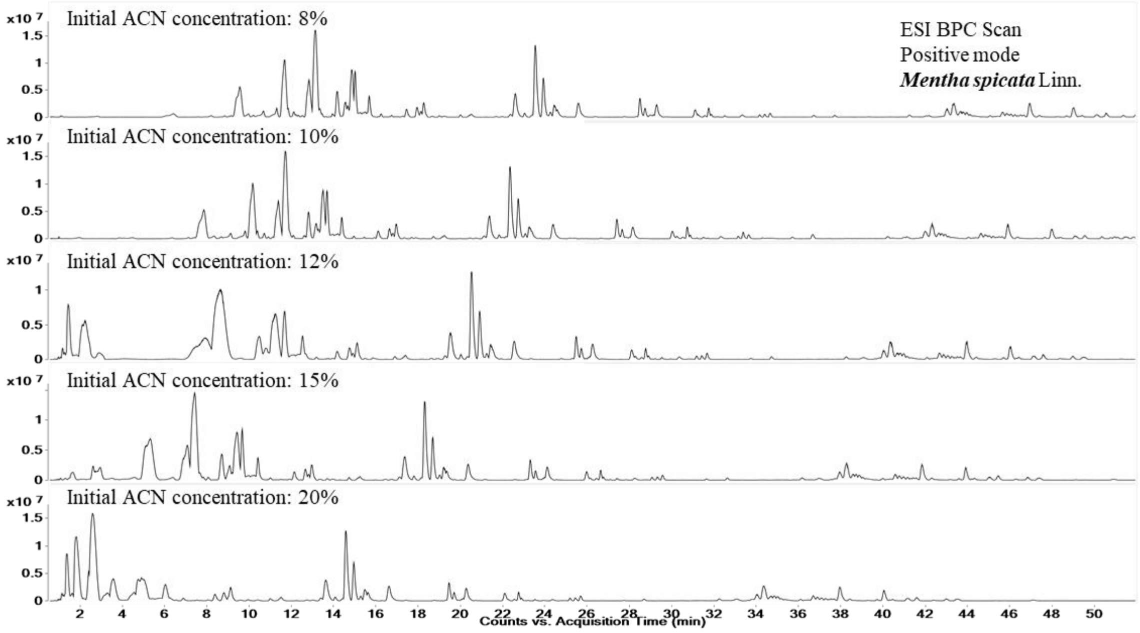

Fig. S2 Base peak chromatogram (BPC) data of fresh samples in different initial ACN concentration

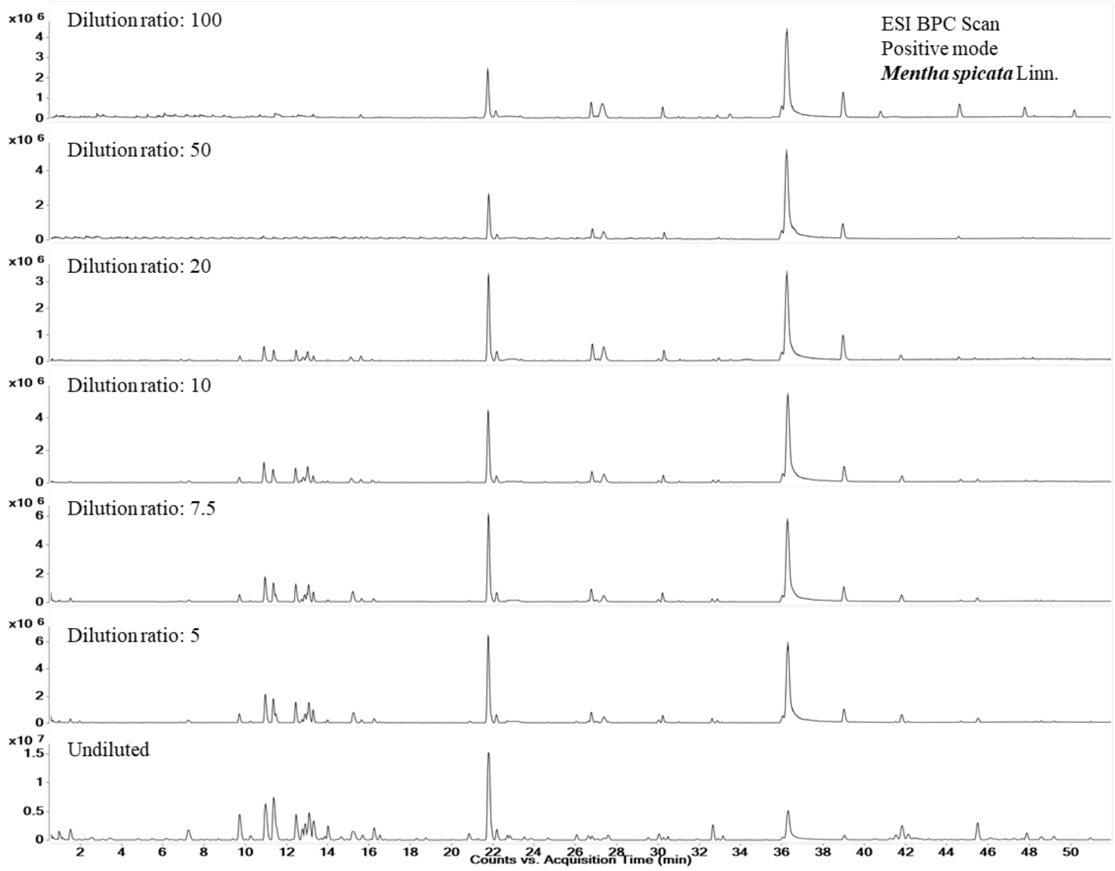

Fig. S3 BPC data of fresh samples in different dilution ratio

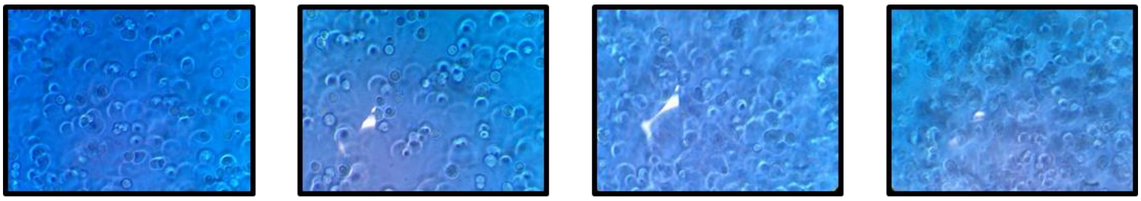

1 d 2 d 3 d 4 d

Fig. S4 Photographs of human embryonic kidney cells in GelMA after encapsulation (1-4 days)
